# RETRACTED: Chen et al. Clinical Effect of Arthroscopic Resection of Extra-Articular Knee Osteochondroma. *J. Clin. Med.* 2023, *12*, 52

**DOI:** 10.3390/jcm12185778

**Published:** 2023-09-05

**Authors:** Peng Chen, Li Shen, Qiong Long, Wei Dai, Xiaocheng Jiang, Canfeng Li, Jianwei Zuo, Jiang Guo, Xintao Zhang

**Affiliations:** 1 Department of Sports Medicine, Peking University Shenzhen Hospital, Shenzhen 518036, China; doc_chenpeng@163.com (P.C.); longqionghao@163.com (Q.L.); qwer4uid@163.com (W.D.); xcjiang_doctor@126.com (X.J.); licanfeng87@163.com (C.L.); zuo0912@163.com (J.Z.); 2 Shenzhen Healthcare Committee Office, Shenzhen 518020, China; francessl@sina.com

Following publication, the authors of “Clinical Effect of Arthroscopic Resection of Extra-Articular Knee Osteochondroma” by Chen et al. [1] contacted the Editorial Office regarding concerns relating to their article. The authors informed the Editorial Office that the same study had been published previously in the *Chinese Journal of Bone and Joint Injury* [2], in Chinese. Adhering to our procedure, an investigation was conducted and confirmed that the overlap included identical figures, as well as the identical analysis of the samples and data interpretation. The Editorial Office and the Editorial Board confirmed that re-publication in English was undertaken without the appropriate authorization from the publisher of the *Chinese Journal of Bone and Joint Injury* and that there were significant differences in the listed authorship of the two manuscripts. This article is therefore retracted.

This retraction was approved by the Editor in Chief of *Journal of Clinical Medicine*. 

The authors agreed to this retraction. 
